# Coriolis Vibratory MEMS Gyro Drive Axis Control with Proxy-Based Sliding Mode Controller

**DOI:** 10.3390/mi13030446

**Published:** 2022-03-16

**Authors:** Derya Ünsal Öztürk, Aydan M. Erkmen

**Affiliations:** 1 Seeker and Guidance Sensors Development Department, Roketsan Missiles Inc., Kemalpaşa Mahallesi Şehit Yüzbaşı Adem Kutlu Sokak No. 21, Elmadağ 06780, Turkey; 2 Department of Electrical and Electronics Engineering, Middle East Technical University (METU), Cankaya, Ankara 06800, Turkey; aydan@metu.edu.tr

**Keywords:** MEMS, gyroscope, sliding mode controller, proxy-based sliding mode controller

## Abstract

MEMS (micro electrical mechanical systems) gyroscopes are used to measure the angular rate in several applications. The performance of a MEMS gyroscope is dependent on more than one factor, such as mechanical imperfections, environmental condition-dependent parameter variations, and mechanical–thermal noises. These factors should be compensated to improve the performance of the MEMS gyroscope. To overcome this compensation problem, a closed-loop control system is one of the solutions. In this paper, a closed-loop control system is implemented. However, other than previously applied methods, a proxy-based sliding mode control approach is proposed, which is a novelty for the control of the MEMS gyroscope drive axis since, to the best of our knowledge, this method has not been applied to gyroscope control problems. Proxy-based sliding mode controllers do not suffer from the chattering phenomenon. Additionally, we do not need an exact system model to implement the control law. In particular, we are investigating, in this paper, the compatibility and performance of a proxy-based sliding mode controller for a closed-loop gyroscope implementation. We show that our proposed method provides robustness against model uncertainties and disturbances and is easy to implement. We also compare the performance of classical sliding mode controllers and proxy-based sliding mode controllers, which demonstrate the evident superiority of the proxy-based controller in our implementation results. Simulation results show that system error and gyroscope total error reduced by 49.52% and 12.03%, respectively, compared to the sliding mode controller. Simulation results are supported with the experimental data, and experimental results clearly demonstrate the superiority of the proxy-based sliding mode controller.

## 1. Introduction

The gyroscope market is growing, powered by increasing applications to missiles, autonomous vehicles, robots, and mobile phones. All of those applications are demanding greater precision day-by-day. Different types of gyros (Ring Laser, Fiber Optic, MEMS) are used in those various applications. Gyros are classified according to bias and bias stability performances: Gyros with bias stability between 0.01 deg/h and 1 deg/h are defined as navigation grade while bias stability between 1 deg/h and 10 deg/h represents tactical grade and greater than10 deg/h represents control/industrial grade. FOG (Fiber Optic Gyroscope) and RLG (Ring Laser Gyro) technology provide navigation and tactical performances. While gyroscope drift dominates the definition of grade, all platforms have their own requirements, such as bandwidth, vibration and shock, power consumption, etc. [1] Although MEMS technology initially provided control/industrial grade performance, today it tries to find a place in more areas by using the advantage of smaller size, low power consumption, and low cost. Therefore, MEMS technology has moved from control grade to tactical and navigation grade. Various methods have been developed by researchers to reach tactical and navigation grade performance. The basis of these methods consists of eliminating the effects of mechanical imperfections, environmental condition-dependent parameter variations, and mechanical–thermal noises that cause performance degradation.

Some of these compensation methods are constructed over hardware such as multiple proof mass usage for better noise performance and bias instability, electrostatic tuning for frequency mismatch, and multi degree of freedom mechanical structures for compensating external effects [2,3,4]. Other methods are based on algorithmic methods using the controller. Instead of modifying sensor design, those methods use controller electronics. Controller-based methods are mostly used to compensate for the effects of changing parameters on the system dynamics due to temperature, environmental factors, etc.; thus, it is aimed to increase the robustness of the sensor. In this study, we recommend the controller-based method. In the literature, there are several controller-based methods.

In the literature, various methods such as the PLL (Phase Locked Loop), adaptive gain control, PID control, and sliding mode control are developed to compensate the effects of mechanical imperfections, thermal effects, packaging stress, etc. The PLL method developed by Wang [5] and Batur et al. [6], compare the performances of a model referenced-based feedback control and sliding mode control to control grade MEMS gyroscopes. The Dynamic Sliding Mode Control method, with a switching function, was developed by Juntano Fei [7]. On the other hand, researchers from the University of California at Berkeley use the adaptive control strategy, RMIT University works on the tri-axial adaptive control algorithm, and Cleveland State University Works on adaptive disturbance rejection for MEMS gyro control [8].

However, it is generally complicated to implement adaptive control methods without high-capacity processors but high-capacity processor usage causes an increase in the size and cost of the sensor and the MEMS industry needs small and low-cost sensor solutions.

At the beginning of this study, performances of classical control methods are investigated. For those methods, knowledge of the gyro model is required. Moreover, due to manufacturing imperfections and parameter changes, designed and manufactured gyroscopes differ from each other. That is why the gyro model, acquired based on assumptions, does not reflect the exact gyro behavior. This difference affects the performance of the mentioned control methods. As a result, the performance of a controller is not sufficient if a gyro model is not exact. Furthermore, if a PID controller is used as the feedback controller, uncertainty of the system decreases the control performance.

Consequently, in this study, a proxy-based sliding mode controller is implemented because of the fact that it does not require an exact gyro model and is resistant to uncertainties. A sliding mode controller is also implemented, not only to compare performances, but also to better understand the basics of PBSMC.

The classical sliding mode controller is insensitive to parameter variations and has complete disturbance rejection. However, the main drawback of the SMC (Sliding Mode Controller) is the chattering caused by the existence of the discontinuous signum function. Although fat boundary layer approaches are used in the design to alleviate the chattering phenomenon [9], they no longer drive the system state to the origin but to a small residual set around the origin [10].

The proxy-based sliding mode control is an extension of the torque-bounded PID (Proportional-Integral-Derivative) position control, and, at the same time, is a modified version of the sliding mode control [11]. The proxy-based sliding mode control is first introduced by Kikuuwe and Fujimoto in [9,12]. The proxy-based sliding mode controller provides smooth, overdamped recovery from large positional errors caused by abnormal events. The main benefits are that small position errors can be remedied by the well-tuned PID controller and large positioning errors can be rapidly recovered owing to the SMC [9]. The idea behind the proxy-based sliding mode control is to attach a virtual object, referred to as a proxy, through a virtual coupling-controlled object. The virtual coupling can perform a PID-type control action to maintain its length as zero [13].

The main contribution of this study is to design a proxy-based sliding mode control for gyro control in order to improve the performance of a MEMS gyro. This paper demonstrates how a proxy-based sliding mode control provides robustness against disturbances and uncertainties beyond the existing approaches in the literature as well as providing smooth, overdamped results and small tracking error.

In the outline of the paper, vibratory gyro theory and the dynamic gyro model are described in Section 2. Section 3 gives our proxy-based sliding mode control design details for overcoming the gyro drive axis control problem. Simulation results and discussions are outlined in Section 4. Experimental studies are presented in Section 5, and conclusions are finally provided in Section 6.

## 2. Coriolis Vibratory MEMS Gyro Basics

An industrial MEMS gyroscope is composed of three main components, which are:

- Sensing Element;
- Read-out Circuit;
- Case.

The sensing element, which measures the angular velocity using the Coriolis effect, is composed of the mass–spring–damper. The readout circuit generates the necessary oscillation/vibration on the drive axis and senses the oscillation on the sense axis. Angular velocity is calculated by the demodulation of the signal on the sense axis. The readout circuit is composed of two parts: the drive mode readout circuit and sense mode readout circuit. To acquire the amplitude of the angular rate, low noise, high resolution, and sensitive detection mode, the readout circuit is required. Generally, controllers are implemented on the drive axis readout circuit to improve gyro measurement accuracy. The case is used to protect the components of the gyroscope from external effects. The external connection interface is also supplied by the case.

Functional block diagrams of the sensing element and details of read-out circuits are provided in the studies of H.J Kwon [14] and Y. Zhao et al. [15].

In this study, we are focused on controlling the motion of the drive axis. Therefore, details about the sensing theory and function of the drive and sense axes are given in Section 2.1.

### 2.1. Vibratory Gyro Theory

**Figure 1:**
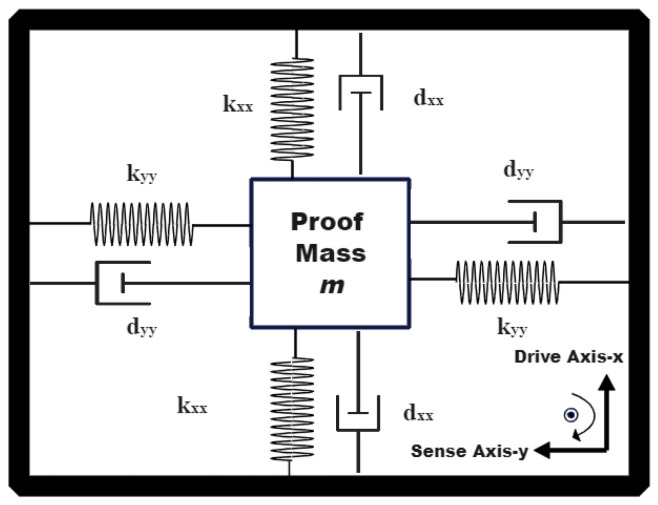
2-DOF Gyroscope Model.

Vibrating MEMS gyroscopes mainly consist of three frames that are mechanically connected by springs, which are the driving frame, sensing frame, and proof mass, simply visualized as a 2-DOF spring-mass-damper system [5] in Figure 1.

Regardless of its type, the main component of a vibratory MEMS gyro is its motion sensitive element. The sensitive element of the Coriolis vibratory gyroscope is called proof mass. The proof mass oscillates on the drive and sense axis that are orthogonal. Primary oscillations are generated on the drive axis intentionally. Because of the Coriolis effect generated by angular velocity, oscillation is generated on the sense axis. Using the oscillation on the sense axis, the angular velocity measured. The Coriolis effect is represented with an imaginary Coriolis force, which is shown as:

(1) $$F_{C}=-2m\vec{\Omega }\times \vec{v}$$

$\vec{\Omega }$: external/axial angular rate;

$\vec{v}$: vector of the primary motion/oscillation.

During the operation, the drive axis is constantly vibrated by means of piezoelectric, electromagnetic, or electrostatic actuation mechanisms to ensure regular movement in the drive axis. The proof mass vibrates in line with the drive and sense frame and transmits rotation-induced energy to the sense mode. The transferred energy is proportional to the magnitude of the rotation and creates a displacement in the sensor axis. This displacement is perceived as rotation by the sense mode of the gyroscope, with piezoelectric, optical, piezo-resistive, or capacitive sensor mechanisms [16].

**Figure 2:**
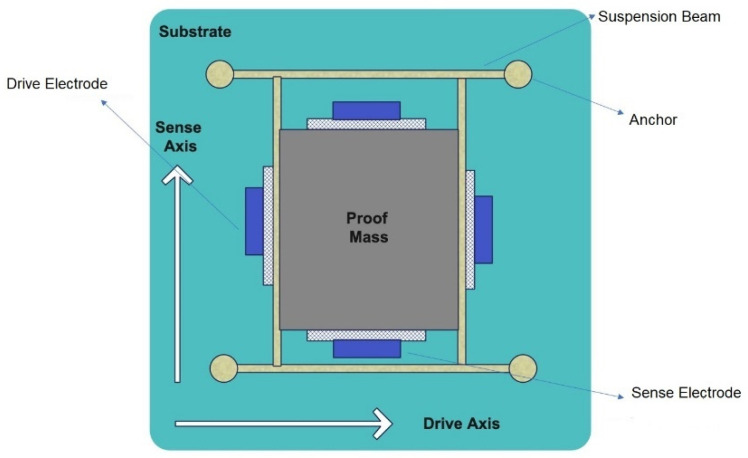
A Generic MEMS Gyro Structure.

A generic MEMS gyro structure is given in Figure 2; a proof mass is suspended above a substrate using a suspension system comprised of flexible beams, anchored to the substrate. There are two sets of electrodes used to excite and detect the output signal. One set of electrodes is needed to excite the drive axis oscillator (desired trajectory/reference vibration) and another set of electrodes detects the sense axis response [17].

The vibration signal applied to the drive axis determines the displacement of the proof mass along the drive axis. To determine the angular rate, demodulation of the proof mass displacement along the sense axis and vibration on the drive axis is used. To increase performance, displacement of the proof mass along the drive axis should be perfect (minimum error between reference vibration and drive axis displacement). However, the presence of unavoidable errors in the manufacturing process, and the influence of the outside ambient temperature, result in the mechanical coupling between the two axes, mechanical–thermal noises, and environmental condition-dependent parameter variations [6,7,18,19]. All these adversary effects degrade the performance of the MEMS gyroscope, so that a closed-loop control system is essential for improving the performance of the MEMS gyroscope through effectively compensating for the mechanical imperfections and the disturbances. Controllers on the drive axis correct the displacement of the proof mass on the drive axis. This directly affects the performance of the gyro. The controller is expected to follow reference displacement with minimum error and delay. In this study, a proxy-based sliding mode control method is implemented, which has not been used for gyro application before.

The controller is designed according to the dynamics of MEMS drive axis dynamics. Additionally, mechanical coupling between two axes, mechanical–thermal noises, and parameter variations effect the performance. Therefore, the dynamics of the sense axis affects the drive axis controller’s performance. Before giving the controller design details, understanding the dynamics of the MEMS gyro is essential. Therefore, the following section gives the dynamic model of a MEMS gyro.

### 2.2. Dynamic Model of a MEMS Gyro

The lumped mathematical model of a vibrating gyroscope is given in (2) and (3) under the assumption that $\Omega_{x}^{2}\approx \Omega_{y}^{2}\approx \Omega_{z}^{2}≅0$ and ${\dot{\Omega }}_{z}\equiv \dot{\Omega }\approx 0$, where Ω is the unknown angular rate:

(2) $$m\ddot{x}+d_{\mathrm{xx}}\dot{x}+k_{\mathrm{xx}}x+d_{\mathrm{xy}}\dot{y}+k_{\mathrm{xy}}y=u_{x}+2m\Omega \dot{y}$$

(3) $$m\ddot{y}+d_{\mathrm{yy}}\dot{y}+k_{\mathrm{yy}}y+d_{\mathrm{xy}}\dot{x}+k_{\mathrm{xy}}x=u_{y}-2m\Omega \dot{x}$$

m is the mass of the proof mass (the mass is restricted to move in the x, y plane), x and y are the coordinates of the proof mass and $k_{\mathrm{xx}},k_{\mathrm{yy}}$ are the spring coefficients. The parameters $d_{\mathrm{xx}},d_{\mathrm{yy}}$ represent the damping and $u_{x},u_{y}$ are the electrostatic driving forces (control input). $2m\Omega \dot{y}$ and $2m\Omega \dot{x}$ are the coupling forces due to the Coriolis effect and $d_{\mathrm{xy}}$ and $k_{\mathrm{xy}}$ are the coupling quadrature and spring errors due to the manufacturing imperfections.

The lumped model can be converted into a parameter-dependent model where these parameters are the quality factor and natural frequency characterizing the gyro:

(4) $$\ddot{x}+\frac{\omega_{\mathrm{nx}}}{q_{x}}\dot{x}+\omega_{\mathrm{nx}}{}^{2}x+\frac{\omega_{\mathrm{nxy}}}{q_{\mathrm{xy}}}\dot{y}+\omega_{\mathrm{nxy}}{}^{2}y=\frac{u_{x}}{m}+2\Omega \dot{y}$$

(5) $$\ddot{y}+\frac{\omega_{\mathrm{ny}}}{q_{y}}\dot{y}+\omega_{\mathrm{ny}}{}^{2}y+\frac{\omega_{\mathrm{nxy}}}{q_{\mathrm{xy}}}\dot{x}+\omega_{\mathrm{nxy}}{}^{2}x=\frac{u_{y}}{m}-2\Omega \dot{x}$$

where

$$\omega_{\mathrm{nx}}=\sqrt{\frac{k_{\mathrm{xx}}}{m}},\;\frac{\omega_{\mathrm{nx}}}{q_{x}}=\frac{d_{\mathrm{xx}}}{m},\;\omega_{\mathrm{ny}}=\sqrt{\frac{k_{\mathrm{yy}}}{m}},\;\frac{\omega_{\mathrm{ny}}}{q_{y}}=\frac{d_{\mathrm{yy}}}{m},\;\frac{\omega_{\mathrm{nxy}}}{q_{\mathrm{xy}}}=\frac{d_{\mathrm{xy}}}{m}$$

In our implementation, simulation models are generated based on (4) and (5). We observe the drive and sense axis characteristics and the closed-loop performance of the gyro control loop. The fundamentals of our applied control methods and controller design steps, namely that for the conventional sliding mode controller and that of our proxy-based sliding mode control design, are outlined in the following section.

## 3. Controller Design

The control problem is to maintain the proof mass m to oscillate in the x-direction at a given desired motion trajectory (desired frequency and amplitude) despite the fact that the motions in the x and y directions are coupled and the angular velocity Ω is unknown.

The desired motion trajectory for the proof mass on the drive axis is given below:

(6) $$x_{d}={A\sin w}_{n}t$$

This section introduces our controller designs as implemented for the gyro drive axis control problem and compared to each other alongside the PID classical control implementation in Section 4.

**Figure 3:**
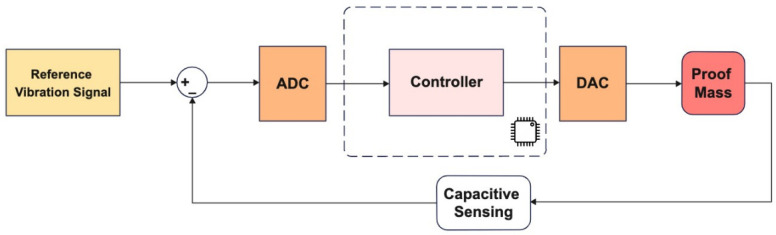
Gyro Drive Axis Controller Application.

The generic control loop architecture for the gyro drive axis control is given in Figure 3. Details of the sliding mode controller implementation is provided in Section 3.1 and the adaptation of the PBSMC (Proxy-Based Sliding Mode Controller) for drive axis control is given in Section 3.2.

### 3.1. Sliding Mode Controller

The sliding mode controller is insensitive to parameter variations and it has complete disturbance rejection. Therefore, it is suitable for gyro proof mass control. It provides robustness against environmental effects (temperature, vibration, etc.) and noise variations, such as mechanical noise and electronic noise.

**Figure 4:**
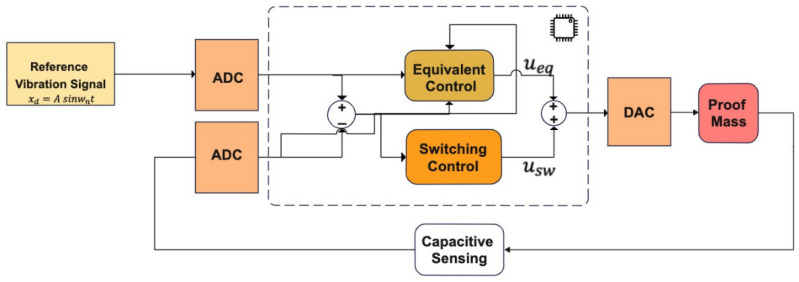
Conventional Sliding Mode Controller.

The conventional sliding mode controller, as we implement it in our gyro controller, is given in Figure 4. The set-point is the desired/reference proof mass drive axis displacement. Additionally, the difference between the desired trajectory and real proof mass trajectory defines the tracking error. The input to the equivalent controller is derived by equating the derivative of the sliding surface to 0: this equivalent controller is a continuous controller to make the sliding surface an eigenmode for the system in question, whereas the switching control is the discontinuous controller determining the convergence to the sliding surface, its input generated by the Lyapunov-based convergence law, the details of which are given below. The switching control input is generated according to the reaching law.

Different types of convergence laws are presented in the literature. Basic types can be listed as the constant rate, the constant plus proportional rate, and the power rate reaching law. The constant reaching law is easily implemented, reduces the chattering effect, and guarantees stability. Therefore, we selected the constant reaching law in this study.

For gyro control application we generate the sliding mode controller as:

(7) $$\ddot{x}+\frac{w_{\mathrm{nx}}}{q_{x}}\dot{x}+w_{\mathrm{nx}}{}^{2}x+\frac{w_{\mathrm{nxy}}}{q_{\mathrm{xy}}}\dot{y}+w_{\mathrm{nxy}}{}^{2}y-2\Omega \dot{y}=\frac{u_{x}}{m}$$

(8) $$s=e+\lambda \dot{e},\;\dot{s}=\dot{e}+\lambda \ddot{e}$$

(9) $$\dot{s}=-\mathrm{Qsgn}\left(s\right)$$

(10) $$u_{\mathrm{eq}}=\frac{m}{\lambda }\left(\lambda {\ddot{x}}_{d}+{\dot{x}}_{d}+\left(2{\lambda \zeta }_{x}w_{\mathrm{nx}}-1\right)\dot{x}+{\lambda w}_{\mathrm{nx}}^{2}x-2{\lambda \zeta }_{\mathrm{xy}}w_{\mathrm{nxy}}\dot{y}-{\lambda w}_{\mathrm{nxy}}{}^{2}y+2\lambda \Omega \dot{y}\right)$$

(11) $$u_{\mathrm{sw}}=-\mathrm{Qsgn}\left(s\right)$$

(12) $$u=u_{\mathrm{eq}}+u_{\mathrm{sw}}$$

where $e=e=x_{d}-x,\dot{e}={\dot{x}}_{d}-\dot{x}$, $\ddot{e}={\ddot{x}}_{d}-\ddot{x}$, $\frac{w_{\mathrm{nx}}}{q_{x}}=2\zeta_{x}w_{\mathrm{nx}}$

s, which is given in Equation (8), defines the sliding surface and λ is a positive constant and is a tuning parameter that defines the slope of the sliding surface and directly affects the system dynamics with fast convergence achieved with high λ. This means that the reaching phase can be shortened by the large amplitude control signal. However, the control signal that can be applied to the system is limited (limited signal sources, processor limits) and high λ causes an overshoot in the system output.

Equation (9) shows the constant rate convergence law where Q is a positive constant that should be large enough to suppress all uncertainties. However, we have limitations on the value of Q. For example, hardware capacities, voltage limitations, and mechanical limitations. Details about the controller parameter determination restrictions are given in the following section.

The equivalent control input is derived by equating the derivative of the sliding surface to 0, which is given in Equation (10), and the switching control input is generated according to the convergence law, given in Equation (11). Equation (12) defines the total additive control input with continuous and discontinuous controllers.

SMC is easily implementable in software and provides fast error convergence. On the other hand, chattering is the main drawback of the sliding mode control. It directly affects the system performance. Chattering is caused by the high-frequency switching of a sliding mode controller exciting unmodeled dynamics [20]. It means that, the effect of the signum function arises due to the uncertainties on the model. There are several methods, such as the boundary layers solution and observer-based solution, which are presented in the literature to reduce chattering. However, these methods can reduce the chattering and do not offer a definite solution. On the other hand, PBSMC, the latest and advanced version of the sliding mode controller, can provide a chattering-free controller solution. Details about the PBSMC is provided in the following section.

### 3.2. Proxy-Based Sliding Mode Controller

Although the sliding mode controller is robust and easy to implement, it also causes a decrease in performance due to the chattering problem. One of the most common methods used to overcome this problem is the proxy-based sliding mode control method.

The Proxy-based Sliding Mode Control is an extension of PID and SMC. This method combines PID control and SMC algebraically to reduce the chattering effect. The PID controller defines low/local dynamics and SMC specifies high/global dynamics of the system. Finally, the PBSMC system provides an overdamped response without sacrificing tracking accuracy.

A proxy (virtual object) is placed between the desired position and real position to implement the proxy-based sliding mode controller to reduce the chattering phenomenon caused by the sign function. The proxy is a virtual object that does not exist in reality and is used for pooling control signals. On the other hand, the PID controller is added between the controlled system and the proxy. The PID controller and SMC are coupled through the proxy.

In this study, as a novel implementation to gyro control, we expect the PBSMC to provide smooth and overdamped solutions under environmental effects (temperature changes, vibration, package stress, etc.) and noise variations, such as mechanical noise and electronic noise.

**Figure 5:**
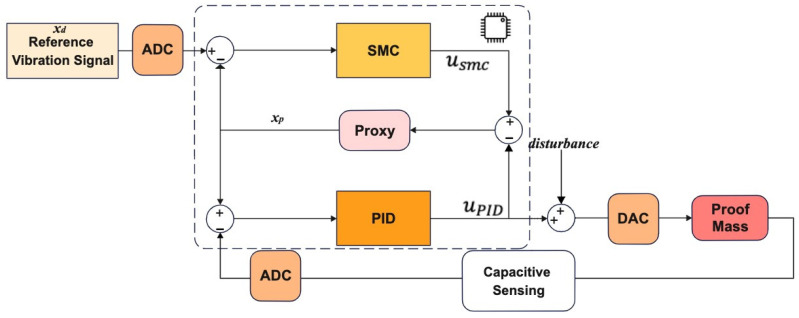
PBSMC (Proxy-Based Sliding Mode Controller) Working Principle.

Our PBSMC implementation is depicted in Figure 5. For our gyro control application, we want to control the displacement of the proof mass on the drive axis. Therefore, motion of the proof mass on the drive axis is our plant. The PID controller, thus, causes an interaction signal $u_{\mathrm{PID}}$ between the plant and proxy. At the same time, the proxy is also controlled by an SMC, which applies $u_{\mathrm{smc}}$, to track the desired reference vibration signal. Small position errors are compensated by the PID controller and large position errors are eliminated by the sliding mode controller.

To start with is the rearrangement of the sliding surface in Equation (8) according to the proxy application.

In the new version of the sliding surface, $x_{d}$ is our desired drive axis displacement and $x_{p}$ is the proxy displacement:

(13) $$s=(x_{d}-x_{p})+\lambda ({\dot{x}}_{d}-{\dot{x}}_{p})$$

(14) $$u_{\mathrm{PID}}=K_{P}(x_{p}-x)+K_{I}\int (x_{p}-x)\mathrm{dt}+K_{D}({\dot{x}}_{p}-\dot{x})$$

Equation (14) provides the PID control signal. Rearranging the sliding surface Equation (8) to merge the PID controller and SMC control signals yields our proxy-based sliding mode control. x is the real displacement of the proof mass on the drive axis:

(15) $$s=(x_{d}-x)+\lambda ({\dot{x}}_{d}-\dot{x})-(x_{p}-x)-\lambda ({\dot{x}}_{p}-\dot{x})$$

(16) $$s=\sigma -\dot{a}-\lambda \ddot{a}$$

where $\sigma =(x_{d}-x)+\lambda ({\dot{x}}_{d}-\dot{x})$ and $a=\int (x_{p}-x)\mathrm{dt}$

The control signal becomes:

(17) $$u_{\mathrm{smc}}=\mathrm{Qsgn}\left(\sigma -\dot{a}-\lambda \ddot{a}\right)$$

(18) $$u_{\mathrm{PID}}=K_{P}\dot{a}+K_{I}a+K_{D}\ddot{a}$$

Considering the proxy, the displacement equation can be derived as ($m_{p}$ is the proxy mass):

(19) $$m_{p}\;{\ddot{x}}_{p}={\;u}_{\mathrm{smc}}-u_{\mathrm{PID}}$$

In practice, Kikuuwe and Fujimoto [11,12] set the proxy mass in (19) to be zero. Then, $u=u_{\mathrm{smc}}=u_{\mathrm{PID}}$ is satisfied. Finally, the PBSMC law is obtained by:

(20) $$u=\mathrm{Qsgn}\left(\sigma -\dot{a}-\lambda \ddot{a}\right)$$

(21) $$u=K_{P}\dot{a}+K_{I}a+K_{D}\ddot{a}$$

(22) $$\ddot{a}=\frac{1}{K_{D}}\;\left(u-K_{I}a-K_{P}\dot{a}\right)$$

Then, to overcome the chattering problem, the discontinuous signum function is replaced with the saturation function with mathematical approximations:

(23) $$u=\mathrm{Qsgn}(\sigma -\dot{a}-\lambda \;\left(\frac{1}{K_{D}}\;\left(u-K_{I}a-K_{P}\dot{a}\right)\right)$$

(24) $$u=\mathrm{Qsgn}\left(\frac{\lambda }{K_{D}}\;\left(\frac{K_{D}}{\lambda \;}\left(\sigma -\dot{a}\right)+K_{I}a+K_{P}\dot{a}-u\right)\right)$$

(25) $$u=\mathrm{Qsat}\left(\frac{1}{Q}\left(\frac{K_{D}}{\lambda \;}\left(\sigma -\dot{a}\right)+K_{I}a+K_{P}\dot{a}\right)\right)$$

where

$$Y=\mathrm{Asgn}\left[B\left(Z-Y\right)\right]\Leftrightarrow Y=A\;\mathrm{sat}\;\left(\frac{Z}{A}\right)$$

$$\mathrm{sat}\left(x\right)=\{\begin{array}{c} \mathrm{sgn}\left(x\right),\;\mathrm{if}\;\left|x\right|>1\\ x,\;\mathrm{if}\;\left|x\right|\leq 1 \end{array}$$

**Figure 6:**
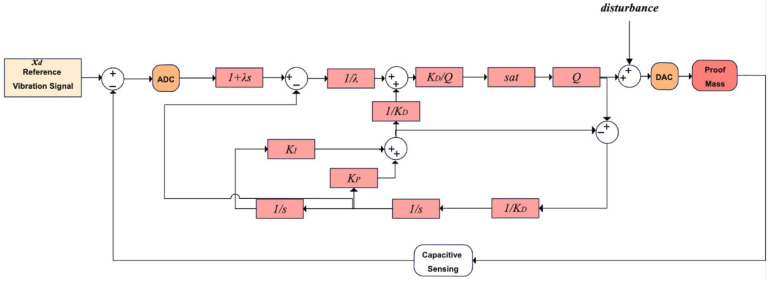
PBSMC for Gyro Drive Axis Control.

Equations (24) and (25) are algebraically equivalent. We can eliminate the chattering effect by using sat instead of sgn. The final and implementable version of PBSMC is illustrated in Figure 6. This model is used in our gyro drive axis control application.

According to the parameter setting (K_P_, K_D_, K_I_, λ), the PBSMC controller can act as a sliding mode controller or PID controller. Situations are summarized in Table 1.

The controller parameters must be determined according to the gyro’s parameters and performance requirements. The first requirement is the minimum steady-state error to achieve high performance. Additionally, we need a fast-settling time for minimum response time. It means that the settling time directly affects the gyro start-up time. If the designer has a limitation for start-up time requirement, controller parameters should be determined according to the limitations. On the other hand, the displacement margin of the proof mass on the drive axis is the critical limit for the overshoot of the controller. Overshoot could not exceed the displacement range of the drive axis.

At the beginning, the controller parameters are determined by simulation studies. After that, they are tuned according to the experimental data to get the best performance. Details of the simulation studies and performance comparisons of PID, SMC, and PBSMC are provided in the following section.

## 4. Simulation Results

A single proof-mass gyro is used in the scope of this study. Gyro parameters, except for the sampling frequency, are the same for both simulation studies and experimental studies. Design parameters of the gyro are given in Table 2.

Simulation scenarios are designed according to our hardware modeling given in Section 2.2 to demonstrate the behavior of a closed-loop drive axis MEMS gyro. A desired driving signal (reference AC vibration signal) is applied as the drive axis input in the simulation. Additionally, disturbance noise is applied to the sensing axis input to simulate the effects of the mechanical–thermal noise and packaging stress. Disturbance noise is modeled as band-limited white noise.

Other parameters about the simulation are summarized in Table 3:

Simulations are run under different conditions to observe the effects of the parameters. For example, input angular rate amplitude, driving signal amplitude, and frequency are changed to understand the method’s sensitivity. On the other hand, the disturbance noise power is changed to observe the robustness.

Controller implementation begins with the PID controller. After that, SMC and PBSMC designs are completed, and the results of the controller solutions are compared.

**Figure 7:**
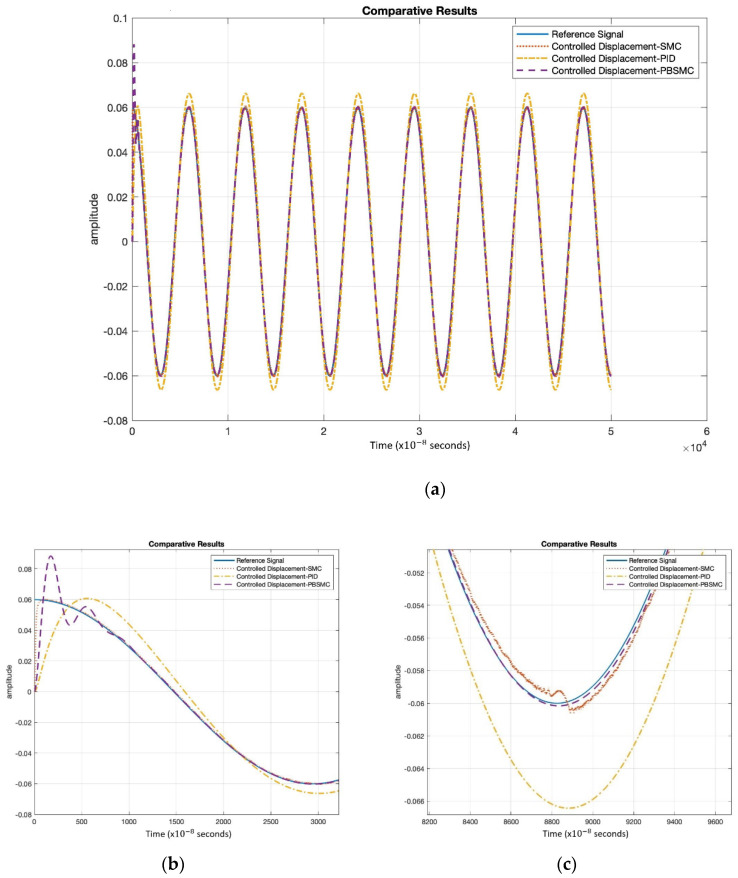
(**a**) PBSMC, SMC, and PID Controller Tracking Performance Comparison, (**b**) Initial Oscillations, (**c**) Steady-State Tracking Performance.

**Figure 8:**
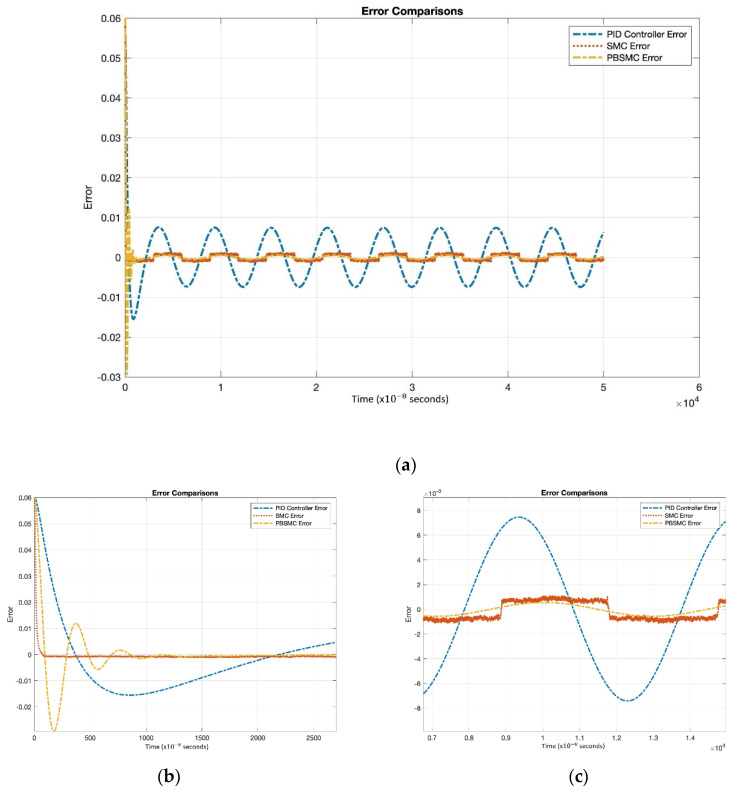
(**a**) Tracking Error Comparison, (**b**) Convergence Performance, (**c**) Steady-State Error Performance.

**Figure 9:**
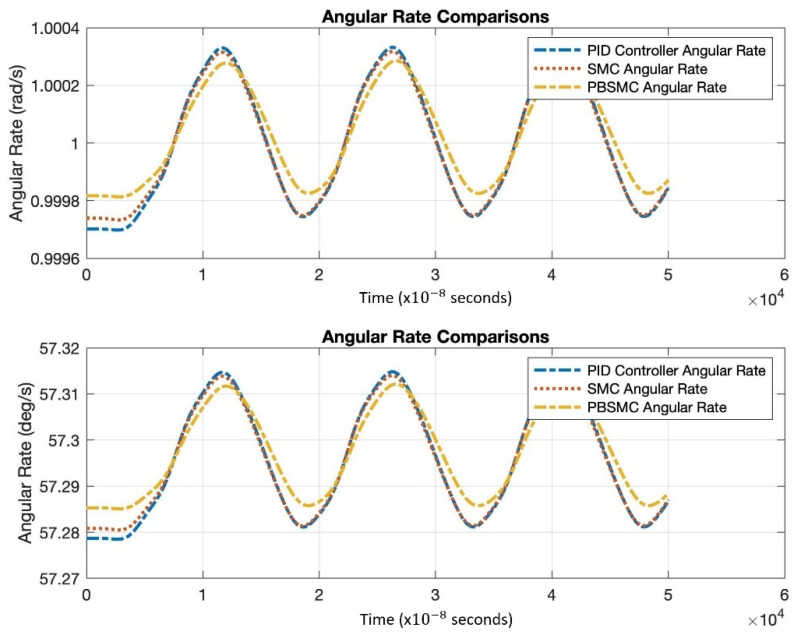
Angular Rate Output Comparison.

Controller parameters are listed in Table 4 and Table 5. The same PID and SMC parameters are used in PBSMC implementation. Simulation results are presented in Figure 7, Figure 8 and Figure 9. Proof mass desired trajectory tracking performances are compared in Figure 7 and controller error performances are given in Figure 8 and angular rate measurement comparisons are shown in Figure 9.

Figure 7 shows that PBSMC provides the best tracking performance. It easily tracks the desired proof mass trajectory. The effect of chattering can be observed for the SMC solution. Additionally, the PID controller can track the trajectory with large error. In Figure 7 and Figure 8, the effect of the signum function is explicitly observed. PBSMC has no chattering effect with the controlled displacement solution.

Figure 8 shows that after the convergence, errors of all the controllers oscillate at around zero. As we expected, oscillation amplitude of the PID is larger than the other controllers. The chattering effect is clearly observed from SMC error. When PID is used without another control method, then the error is not zeroized and it oscillates. On the other hand, using SMC, the error gets close to zero but performance decreases because of chattering. The PID controller defines low/local dynamics and SMC specifies high/global dynamics of the system. Because of this condition, using PBSMC, we can reach minimum error and eliminate the chattering effect.

When error convergence times of the controller are compared, SMC converges from 50 × $10^{-8}$ s, PBSMC converges from 880 × $10^{-8}$ s, and PID converges from 110 × $10^{-8}$ s. The effect of convergence time over gyro start-up time is negligible. Moreover, high convergence time has no negative effect on the angular rate data that is acquired using the output of the sense axis. The reason why, is that the group delay of LPF, used for demodulation, is bigger than any convergence time.

Figure 9 shows the angular rate output, which is acquired by the demodulation and filtering of the sense axis output. All the controller angular rate measurement solutions oscillate; however, PBSMC has the lowest peak-to-peak amplitude. The PBSMC solution is not only less noisy but its drive axis control error is also so small that its effect can be seen on the angular rate output. All angular rate measurements oscillate around the input angular rate 1 rad/s (57.2958 deg/s) and PBSMC has less error than the others. This overriding performance directly affects the navigation performance.

Moreover, SMC and PBSMC are less sensitive to frequency and amplitude change; however, track performance of PID decreases with frequency increase.

Table 6 also shows the superiority of PBSMC numerically. As expected, steady-state RMS error and tracking delay performance is better than the other controllers. There is an oscillation on the initialization of PBSMC. Therefore, RMS error of PBSMC is greater than the SMC. The amplitude of this oscillation can be reduced with the adjustment of the PID controller coefficients. Error convergence times are provided for comparison. Differences in this level do not affect the sensor performance.

The robustness performance of controllers is simulated by changing the amplitude of the noise. With the effect of increasing noise, track performance of the PID drastically decreases. This directly affects the linearity of the gyro. SMC performance is not affected by amplitude change. When all of them are compared, PBSMC tracking performance overrides all other methods, shown in Table 7.

Total angular rate measurement error is summarized in Table 8.

## 5. Experimental Results

**Figure 10:**
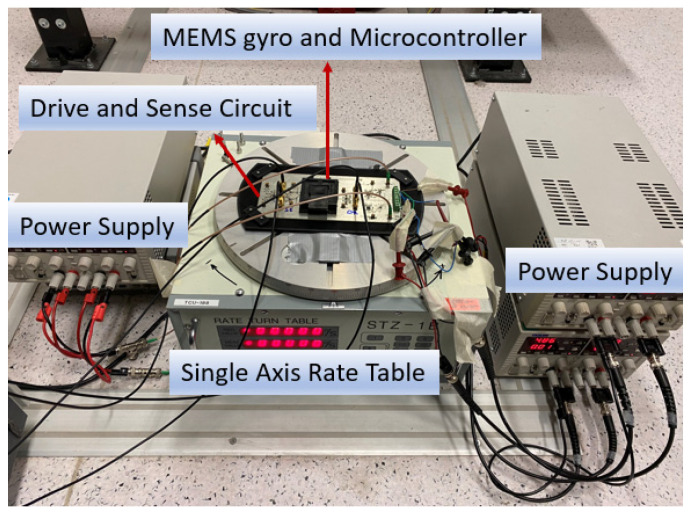
Experimental Setup.

In this section, we present results from the rate table experimental tests performed to verify simulation results and controller performances. A single axis and single proof mass MEMS gyro prototype is used in the experimental studies and experimental setup is presented in Figure 10. Design parameters of the gyro are given in Table 2.

For the test setup, the MEMS prototype is driven with the circuit running on the drive axis. For the tests, a single axis rate table is used. Developed controllers are programmed over a low-cost microcontroller. Angular rate measurements are gathered by demodulation of the data on the sense axis. For the tests, the Agilent MSO9254A Mixed Signal Oscilloscope (Analog Signals are sampled with 500 Mhz on oscilloscope probe and stored in the oscilloscope memory) is used with a 50 MHz sampling rate to log the required data. Tests are implemented at 0.1745 rad/s (10 deg/s) and 0.3490 rad/s (20 deg/s) to understand the angular rate error performance under different angular rates.

Applications held with experimental data show that PBSMC has the best performance, which supports the simulation data. PID performance shows worse performance than the simulation. This is because of the fact that the input signal is noisier than the simulation data. Moreover, the parameters which cause uncertainty in the system are more effective in the experiment than the simulations. The effect of noisy reference signals is not seen in the outputs of PBSMC and SMC controllers. In conclusion, PID performance is worse than the simulation in the experiment: however, SMC and PBSMC performances are close to the simulations. Moreover, because of the LPF applied to the demodulated data, the effect of the noise on the angular rate cannot be seen in the experiment.

**Figure 11:**
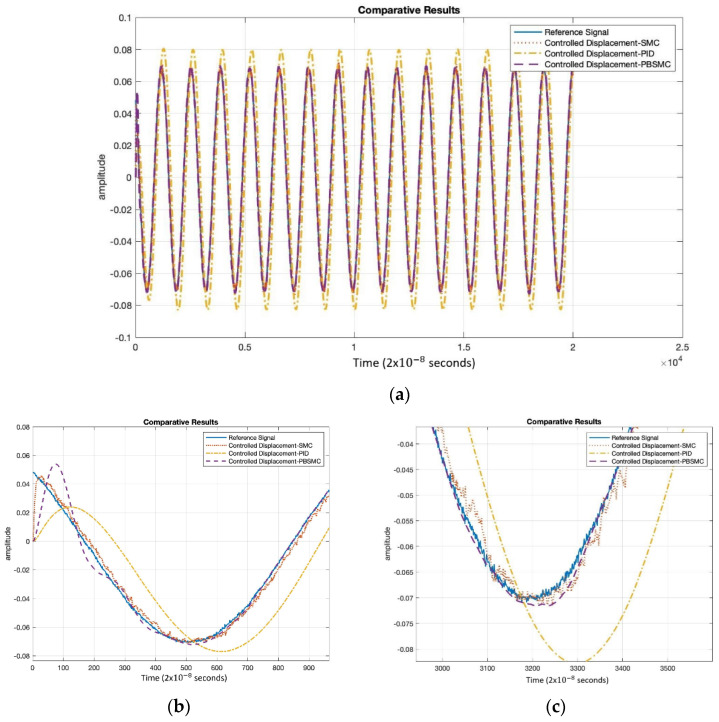
(**a**) Experimental Controller Tracking Performance Comparison, (**b**) Initial Oscillations, (**c**) Steady-State Tracking Performance.

**Figure 12:**
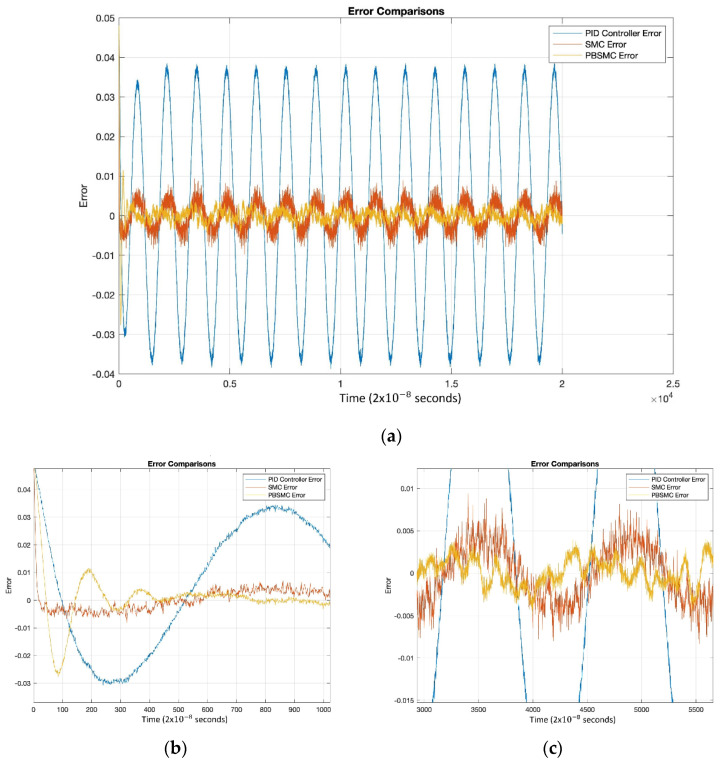
(**a**) Experimental Controller Error Performance Comparison, (**b**) Convergence Performance, (**c**) Steady-State Error Performance.

**Figure 13:**
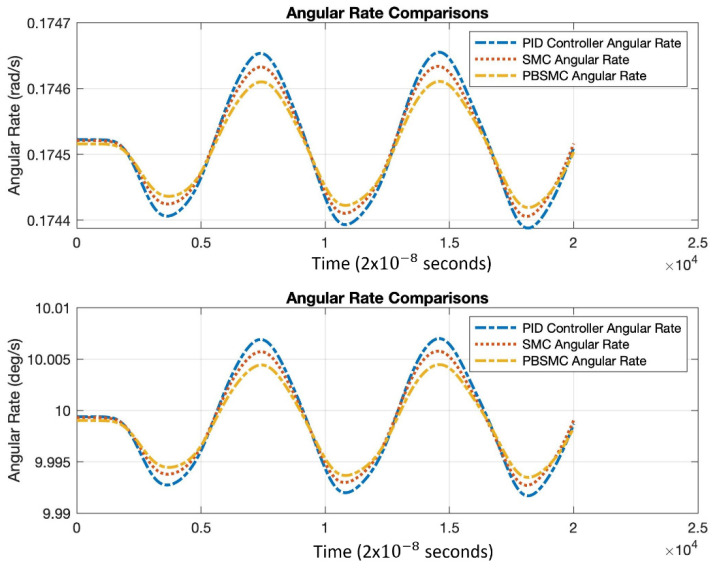
Angular Rate Measurement Comparison.

The result of the experiments proves the data on simulation. PBSMC performed is better than other control methodologies, not only for desired trajectory performance, but also angular rate measurement performance. Experimental PBSMC initial oscillation data is slightly different from the simulation. When angular rate measurement is investigated, PBSMC overrides other control methods for angular rate measurement, which oscillates at the approximately applied 10 deg/s rate. Test results are shown in Figure 11, Figure 12 and Figure 13 and summarized in Table 9 and Table 10.

Figure 11 shows that PBSMC performs better than the other control. The chattering effect on the SMC solution is clearly observable. On the other hand, the overall performance of the PID controller has worsened.

The decrease in the performance of the PID controller is better understood with Figure 12. Additionally, there are degradations in the performance of SMC, but the amount degradation is negligible when compared with PID performance. This proves the robustness of SMC and PBSMC.

According to Figure 13, similar to simulation studies, PBSMC provides the least erroneous angular velocity measurement.

When simulation results and experimental results are compared (Table 6 and Table 9), the error level for the PID is higher than the simulation outputs. In contrast, PBSMC and SMC error levels are close to the simulation results. This proves that PBSMC and SMC performance is not affected by the uncertainties in the model.

The result of the experiments proves the data on simulation. PBSMC performs better than other control methodologies, not only for the desired trajectory performance, but also angular rate measurement performance. Experimental PBSMC initial oscillation data is slightly different from simulation. When angular rate measurement is investigated, PBSMC overrides other control methods for angular rate measurement, which oscillates at the approximately applied 10 deg/s angular rate. Additionally, change in the input angular rate does not affect the controller performance.

## 6. Conclusions

In this paper, we proposed control methods for the Coriolis MEMS gyro drive axis. We tried to get better angular rate measurement performance by controlling the proof mass movement. A proxy-based sliding mode controller is proposed as a MEMS gyro controller, and a sliding mode controller and PID controller is implemented to understand the superiority of PBSMC. According to the simulation and experimental data, tracking error is reduced and overall gyro performance increases with the proxy-based sliding mode controller. It is easily implemented, does not require high process capabilities, and it is robust against model uncertainties. For this reason, PBSMC is suitable for MEMS gyro applications.

PID is a well-known and easily implemented control method; however, its performance is directly affected by noise and uncertainties when implemented on these types of applications. Moreover, PID performance is worse than other control methods under temperatures different than room temperature. SMC also performs worse than PBSMC because of the chattering phenomena.

Simulation data and experimental data shows SMC and PBSMC control methods are robust methods; however, PBSMC has better performance because of the SMC chattering effect.

As a result of the studies, it is evaluated that the proposed method is suitable and applicable for gyro applications. With the application of the related method, it is aimed to achieve a significant increase in the performance of low-cost sensors. This significant increase directly improves the platform’s navigation performance.

Finally, the proposed method, the proxy-based sliding mode controller, can be easily used in gyroscopes with resonating mass, regardless of its structure.

Tests under different temperature and vibration levels can be listed as the future work of this study. Robustness of PBSMC will be proven again with these studies.

## Figures and Tables

**Table 1:** PBSMC Behaviors According to Parameters.

Parameter Values	Result
K_P_ → ∞	Sliding Mode Controller
λ = K_D_/K_P_, K_I_ = 0	PID Controller

**Table 2:** MEMS Gyro Parameters.

Parameter	Value
Mass (m)	$0.57\times 10^{-8}$ kg
K_xx_	235.8 N/m
C_xx_	$0.429\times 10^{-6}$ N s/m
K_yy_	240.3 N/m
C_yy_	$0.687\times 10^{-3}$ N/m
K_xy_	5 N/m
C_xy_	$0.429\times 10^{-6}$ N s/m

**Table 3:** Simulation Parameters.

Parameter	Value
Input Angular Rate	1 rad/s (57.2958 deg/s)
Desired Driving Signal Amplitude	600 mV
Desired Driving Signal Frequency	17,000 Hz
Sampling Period	$1\times 10^{-8}$ sn
Disturbance Noise Power	0.3

**Table 4:** PID Controller Parameters.

Parameter	Value
K_I_	5878
K_P_	2.0789
K_D_	1.6038 $\times \;10^{-4}$

**Table 5:** Sliding Mode Controller Parameters.

Parameter	Value
Q	900
λ	100

**Table 6:** Error Performance and Convergence Time for Controllers.

	PID	SMC	PBSMC
RMS Error	0.0070	0.0014	0.0031
Steady State RMS Error	0.0052	$7.8754\times 10^{-4}$	$3.9752\times 10^{-4}$
Error Convergence Time	$376\times 10^{-8}$ s	$93\times 10^{-8}$ s	$870\times 10^{-8}$ s

**Table 7:** Error Performance Comparison for Different Noise Levels.

	PID	SMC	PBSMC
SS RMS Error—0.5 Noise power	0.0156	$7.8621\times 10^{-4}$	$5.2020\times 10^{-4}$
SS RMS Error—0.8 Noise power	0.0196	$7.8532\times 10^{-4}$	$5.8340\times 10^{-4}$
SS RMS Error—1.5 Noise power	0.0267	$7.8707\times 10^{-4}$	$7.0998\times 10^{-4}$

**Table 8:** Angular Rate Total Error Comparison.

	PID	SMC	PBSMC
Maximum Error	−67.9580 deg/h	−64.9040 deg/h	−57.2425 deg/h

**Table 9:** Error Performance and Convergence Time Comparison for Experimental Data.

	PID	SMC	PBSMC
RMS Error	0.0257	0.0034	0.0029
Steady-State RMS Error	0.0259	0.0033	0.0014
Error Convergence Time	$110\times 10^{-8}$ s	$50\times 10^{-8}$ s	$880\times 10^{-8}$ s

**Table 10:** Angular Rate Total Error Comparison for Experimental Data.

	PID	SMC	PBSMC
Maximum Error for 10 deg/s	31.6897 deg/h	27.3923 deg/h	22.7411 deg/h
Maximum Error for 20 deg/s	32.2484 deg/h	28.5214 deg/h	23.6921 deg/h

## Data Availability

Not applicable.
